# Determinants of complementary feeding practices among Nepalese children aged 6–23 months: findings from demographic and health survey 2011

**DOI:** 10.1186/1471-2431-13-131

**Published:** 2013-08-28

**Authors:** Vishnu Khanal, Kay Sauer, Yun Zhao

**Affiliations:** 1 School of Public Health, Curtin University, Perth, Western Australia, Australia; 2 Centre for Behavioural Research in Cancer Control, Curtin University, Perth, Western Australia, Australia

## Abstract

**Background:**

The adoption of inappropriate feeding practices is one of the reasons for under nutrition in Nepal and elsewhere. The objective of this study was to describe the rate of and identify the factors associated with providing the World Health Organization (WHO) recommended infant feeding practices of minimum dietary diversity, minimum meal frequency and minimum acceptable diet in Nepal amongst young children between 6–23 months in 2011.

**Methods:**

Data from Nepal Demographic and Health Survey (NDHS) 2011 was used. Prevalence of minimum dietary diversity, minimum meal frequency and minimum acceptable diet was obtained by using descriptive statistics. A Chi-square test (χ^2^) followed by multiple logistic regression analyses were used to determine the adjusted effect of potential factors on the outcome variables.

**Results:**

Of the 698 children aged 6–23 months; while 535 (76.6%) received the minimum meal frequency, only 212 (30.4%) children received the minimum dietary diversity, and 185 (26.5%) received an acceptable diet. Children of older mothers (>35 years); educated mothers and fathers; and mothers from all the development regions except the Mid-western region were more likely to have been provided with the recommended dietary diversity. Children of mothers who had attended ≥4 antenatal visits and who lived in the Eastern region were more likely to provide their child with the recommended meal frequency. Children of mothers, who attended ≥ 4 antenatal visits, were educated and whose fathers had at least a secondary education were more likely to meet the recommended acceptable diet standards.

**Conclusion:**

Young children aged less than two years in Nepal are at risk for not meeting the WHO recommended infant feeding standards given that only about one in three children were provided with the recommended dietary diversity and acceptable diet. This finding suggests that the majority of children are at risk of under nutrition. An appropriate mix of health education and food supplements could be a feasible option for Nepal to improve the number of children who meet the recommended infant feeding guidelines, reduce under nutrition and improve the survival rates of young children.

## Background

In 2006 an estimated 9.5 million children died worldwide before the age of five; of which 35% were due to under nutrition [1,2]. The major measures of under nutrition in developing countries include stunting, wasting, and micronutrient deficiency [2]. Children aged 6–23 months are at a greater risk to suffer from under nutrition. It is during this period that a child moves from mother’s milk to solid foods as its primary source of nutrition. When introduced to solid foods, the child may suffer from indigestion, infection, insufficient food or a combination of these. This can result in childhood under nutrition. Like many developing countries, wasting and stunting have long been problems in Nepal. There has been little improvement in the nutritional status of children over the past decade 2001–2011 [3,4]. The proportion of children underweight (weight <2 SD below the mean) was 10% during 2001 and had increased to 13% in 2011. Stunting (defined as height for age <2 SD below the mean) was 43% in 2001 and 41% in 2011 [3,4]. Given that under five mortality is 54 per 1000 live births, the existing high proportion of under nutrition makes it complicated to reduce under five mortality rates [4]. Under nutrition in early life has further long-term impacts when the child becomes an adult leading to an intergenerational effect and causing loss of productivity [5]. Providing an age appropriate and adequate diet is a proven measure to reduce under nutrition among children.

Complementary feeding practice is a significant factor that determines the nutritional status of children. Suboptimal infant feeding practices are the major reasons for childhood under nutrition in developing countries including Nepal. The transition period from exclusive breastfeeding to two years is a critical window for optimal growth and development of the child. During this period, appropriate, safe, adequately nourished and frequent feeding is essential. The caregiver should know what, how, and when to give appropriate food. Unknowingly, the food provided to a child might be too high or too low in some nutrients, the diversity of food might be adequate or inadequate, and micronutrient content including iron could be lower than required if they rely on certain food items such as cow’s milk and simple porridge [6]. The World Health Organization (WHO) has developed eight core infant and young child feeding indicators to monitor and to guide the feeding practices of young children [7]. WHO core indicators include: (1) early initiation of breastfeeding; (2) exclusive breastfeeding under six months; (3) continued breastfeeding for one year; (4) the introduction of solid, semi-solid or soft foods; (5) minimum dietary diversity; (6) minimum meal frequency; (7) minimum acceptable diet; and (8) consumption of iron rich or iron fortified foods.

Infant and young child feeding indicators are relatively new and; little has been explored in Nepal. Only one study has reported these indicators using the 2006 NDHS data [8]. The study reported that 82% of the children aged 6–23 met minimum meal frequency while 34% met diversity and only 32% had had an acceptable diet [8]. However, the study [8] excluded children who were not breastfed when examining the minimum acceptable diet indicator because not all information was collected in 2006. The 2011 NDHS did include information for non-breastfed children which enable their information to be included in the analysis giving more accurate estimates of feeding practices. Updated knowledge of feeding practices will assist the national nutrition program to better monitor the changes in the feeding practices and design interventions to increase the recommended feeding practices and thereby contribute in reducing under nutrition in Nepal and elsewhere. Utilizing the NDHS 2011 data, this study aimed to (i) describe the rate of providing minimum dietary diversity, minimum meal frequency and minimum acceptable diet amongst young children aged 6–23 months in 2011 and (ii) identify the factors associated with minimum dietary diversity, minimum meal frequency and minimum acceptable diet amongst young children aged 6–23 months.

## Methods

### Data source

The NDHS 2011 interviewed a total of 12,674 women and 4,121 men [4]. The response rate was 95.3%. Three sets of questionnaire were used to collect different types of information: (1) Household level information; (2) Women’s information from women 15–49 years; and (3) Men’s information from men 15–49 years [4]. Information from the child dataset was used in this study. The child dataset contains the information from these three questionnaires relevant to child health.

### Variables

The WHO recommended infant feeding guidelines are used for defining the outcome variables.

Food groups: NDHS collects the information on several food items provided to young children within 24 hours preceding the survey [4]. These food items were re-grouped into seven food groups according to WHO infant feeding guideline [9]: (i) grains, roots and tubers; (ii) legumes and nuts; (iii) dairy products; (iv) flesh foods; (v) eggs; (vi) vitamin A rich fruits and vegetables; and (vii) other fruits and vegetables.

Minimum dietary diversity: the proportion of children who ate at least 4 or more varieties of foods from the seven food groups in a 24 hour time period [7-9].

Minimum meal frequency: the proportion of children who received complementary foods the minimum recommended number of times in 24-hours. For breastfed children, the frequency should be at least 2 times for 6–8 months, and at least 3 times for 9–23 months of age. For non-breastfed children, it should be at least 4 times in last 24 hours [9]. Meal frequency for non-breastfed children counts the number of times the children were given milk products (formula milk, milk or yoghurt) as a separate food group. These items are not included in the count for breastfed children.

Minimum acceptable diet: a composite indicator of minimum dietary diversity and minimum meal frequency. When a currently breastfed child meets both the minimum diversity and the minimum meal frequency, the child is considered to have met the WHO recommended minimum acceptable diet. However, this indicator is slightly different for the non-breastfed child. Dietary diversity is calculated by using six food groups (excluding dairy products) at least four times a day and combining milk related products (formula milk, milk or yoghurt) at least two times in the day. When both of these criteria were met and the conditions for minimum meal frequency for non-breastfed children were met, the child was considered to be receiving a minimum acceptable diet [9]. The independent variables in this study are based on the categories provided in the NDHS [4], NDHS based studies from Nepal [8,10] and other similar South Asian studies [5,11-14].

### Statistical analysis

The three infant feeding indicators (complementary feeding), minimum dietary diversity, minimum meal frequency and minimum acceptable diet were reported in this study. The prevalence of complementary feeding practices (with regards to key practices) was obtained by using descriptive statistics (frequency distribution). Missing values were coded as food not provided. By doing so, the false positive status of prevalence (feeding practice) was minimized. Categorical variables of interest associated with, minimum meal frequency, minimum dietary diversity and minimum acceptable diet were determined by using a Chi-square test (χ^2^). The association between the significant factors reported in univariate analysis was further examined by using multiple logistic regression after controlling for potential confounders in the model. The stepwise backward elimination procedure was used in the multiple logistic regression. Crude and adjusted odds ratio (aOR); and their 95% confidence interval (CI) were reported. A p-value < 0.05 was considered statistically significant. The statistical analyses were conducted using Statistical Package for Social Science, Advanced statistics, Release 19.0 (SPSS for windows, SPSS Inc., Chicago, IL, USA).

### Ethics

The NDHS surveys were approved by Nepal Health Research Council, Nepal and ICF Macro Institutional Review Board in Calverton, Maryland, USA. The NDHS obtained written consent from the respondents. Mothers provided consent for her children to provide the information. For this study, Curtin University Human Research Ethics Committee ethics approval [protocol approval –SPH-16-2012] was obtained. Permission from Macro International (research agency) was obtained for the use of data.

## Result

### Characteristics of the sample

Table 1 presents the characteristics of mother - child pairs. A total of 698 children aged 6–23 months were enrolled in the 2011 survey with the majority of their mothers (68.6%) aged between 20–29 years. A small proportion (11.2%) of mothers was teenagers. Only 20% of mothers were from urban areas and 19.6% were from the mmountainous areas. The proportion of mothers with no formal education was 41.3% in comparison to only 19.3% of fathers with no education. The majority of mothers (59%) were involved in agriculture and only 12.8% in paid work. Most of the fathers (71.5%) were involved in non-agricultural occupations. Half of the respondents belonged to poor families. The majority (85%) used cooking fuels which were highly polluting in nature (Table 2).

**Table 1:** Rate (%) of meeting minimum dietary diversity among children of age 6–23 months by demographic and socioeconomic characteristics, Nepal 2011 (N=698)

**Factor**	**Category**	**Total** **N [%]**^**#**^	**MDD** **n [%]**	**p value**	**MMF** **n [%]**	**p value**	**MAD** **n [%]**	**p value**
**Mother’s age at pregnancy(n= 698)**				0.061		0.525		0.014
(in years)								
	15–19	78 [11.2]	22 [28.2]		57 [73.1]		21 [26.9]	
	20–29	479 [68.6]	159 [33.2]		371 [77.5]		141 [29.4]	
	30–34	83 [11.9]	16 [19.3]		60 [72.3]		11 [13.3]	
	>=35	58 [8.3]	15 [25.9]		47 [81.0]		12 [20.7]	
**Place of residence(n=698)**				0.004		0.690		0.025
	Urban	138 [19.8]	56 [40.6]		104 [75.4]		47 [34.1]	
	Rural	560 [80.2]	156 [27.9]		431 [77.0]		138 [24.6]	
**Development region(n=698)**				0.003		0.009		0.007
	Eastern	165 [23.6]	62 [37.6]		142 [86.1]		55 [33.3]	
	Central	157 [22.5]	41 [26.1]		116 [73.9]		35 [22.3]	
	Western	100 [14.3]	36 [36.0]		79 [79.0]		30 [30.0]	
	Mid -western	167 [23.9]	34 [20.4]		117 [70.1]		30 [18.0]	
	Far-western	109 [15.6]	39 [35.8]		81 [74.3]		35 [32.1]	
**Ecological region(n=698)**				0.097		0.006		0.030
	Mountain	137 [19.6]	41 [29.9]		105 [76.6]		33 [24.1]	
	Hill	283 [40.5]	98 [34.6]		233 [82.3]		90 [31.8]	
	Terai/Plain	278 [39.8]	73 [26.3]		197 [70.9]		62 [22.3]	
**Mother’s education(n=698)**				<0.001		0.005		<0.001
	No education	288 [41.3]	46 [16.0]		203 [70.5]		40 [13.9]	
	Primary	131 [18.8]	31 [23.7]		100 [76.3]		25 [19.1]	
	Secondary	236 [33.8]	11 [47.0]		195 [82.6]		100 [42.4]	
	Higher	43 [6.2]	24 [55.8]		37 [86.0]		20 [46.5]	
**Father’s education(n=698)**				<0.001		0.003		<0.001
	No education	135 [19.3]	11 [8.1]		91 [67.4]		9 [6.7]	
	Primary	155 [22.2]	33[21.3]		112 [72.3]		26 [16.8]	
	Secondary	334 [47.9]	13[39.2]		275 [82.3]		118 [35.3]	
	Higher	74 [10.6]	37[50.0]		57 [77.0]		32 [43.2]	
**Mother’s occupation(n= 698)**				<0.001		0.592		0.002
	Not working	197 [28.2]	56 [28.4]		146 [74.1]		50 [25.4]	
	Agriculture	412 [59.0]	113[27.4]		319 [77.4]		98 [23.8]	
	Working (paid)	89 [12.8]	43 [48.3]		70 [78.7]		37 [41.6]	
**Father’s occupation(n=698)**				0.132		0.039		0.065
	Agriculture	186 [26.6]	46 [24.7]		134 [72.0]		38 [20.4]	
	Non agriculture	499 [71.5]	161[32.3]		388 [77.8]		142 [28.5]	
	Others	13 [1.9]	5 [38.5]		13 [100]		5 [38.5]	
**Ethnicity(n=698)**				0.002		0.998		0.020
	Relatively advantaged	324 [46.4]	120 [37.0]		248 [76.5]		102 [31.5]	
	Relatively disadvantaged (Janjati)	241 [34.5]	61 [25.3]		185 [76.8]		55 [22.8]	
	Relatively disadvantaged (Dalit)	133 [19.1]	31 [23.3]		102 [76.7]		28 [21.1]	
**Religion(n=698)**				0.035		0.575		0.090
	Hindu	592 [84.8]	189 [31.9]		456 [77.0]		164 [27.7]	
	Others	106 [15.2]	23 [21.7]		79 [74.5]		21 [19.8]	
**Sex of child(n=698)**				0.163		0.900		0. 155
	Male	331 [47.4]	109 [32.9]		253 [76.4]		96 [29.0]	
	Female	367 [52.6]	103 [28.1]		282 [76.8]		89 [24.3]	
**Sex of household head(n=698)**				0.697		0.018		0.972
	Male	520 [74.5]	160 [30.8]		387 [74.4]		138 [26.5]	
	Female	178 [25.5]	52 [29.2]		148 [83.1]		47 [26.4]	
**Wealth index(n= 698)**				<0.001		0.341		<0.001
	Poorest	205 [29.4]	37 [18.0]		156 [76.1]		34 [16.6]	
	Poor	139 [19.9]	36 [25.9]		102 [73.4]		29 [20.9]	
	Middle	131 [18.8]	36 [27.5]		96 [73.3]		31 [23.7]	
	Richer	124 [17.8]	55 [44.4]		99 [79.8]		51 [41.1]	
	Richest	99 [14.2]	48 [48.5]		82 [82.8]		40 [40.4]	
**Type of cooking fuel(n=698)**				<0.001		0.022		<0.001
	Relatively non polluting	103 [14.8]	54 [52.4]		88 [85.4]		47 [45.6]	
	Relatively highly polluting	595 [85.2]	158 [26.6]		447 [75.1]		138 [23.2]	

# The number of missing values may vary for each variable. The percentages presented are valid percentages. *MDD* Minimum dietary diversity, *MMF* Minimum meal frequency, *MAD* Minimum acceptable diet.

**Table 2:** Rate of meeting minimum dietary diversity among children of age 6–23 months by health related characteristics, Nepal 2011 (N=698)

**Factor**	**Category**	**Total** **N [%]**^**#**^	**MDD** **n [%]**	**p value**	**MMF** **n [%]**	**p value**	**MAD** **n [%]**	**p value**
**Birth order (n=698)**				<0.001		0.106		<0.001
	First	252 [36.1]	96 [38.1]		202 [80.2]		85 [33.7]	
	Second or third	306 [43.8]	95 [31.0]		234 [76.5]		84 [27.5]	
	Fourth or more	140 [20.1]	21 [15.0]		99 [70.7]		16 [11.4]	
**Birth interval(n=698)**				0.004		0.225		0.005
	No previous birth	252 [36.1]	96 [38.1]		202 [80.2]		85 [33.7]	
	< 24 months	99 [14.2]	27 [27.3]		72 [72.7]		26 [23.2]	
	>=24 months	347 [49.7]	89 [25.6]		261 [75.2]		77 [22.2]	
**Timing of pregnancy(n=698)**				<0.024		0.057		0.019
	Wanted then	514 [73.6]	165 [32.1]		401 [78.9]		145 [28.5]	
	Wanted later	87 [12.5]	29 [33.3]		62 [68.9]		25 [27.8]	
	Wanted no more	97 [13.9]	18 [18.6]		72 [72.0]		15 [15.0]	
**Anaemia level of mother(n= 688)**				0.006		0.013		0.009
	Moderate	38 [5.5]	5 [13.2]		26 [68.4]		5 [13.2]	
	Mild	201 [29.2]	51 [25.4]		142 [70.6]		42 [20.9]	
	Not anaemic	449 [65.3]	152 [33.9]		360 [80.2]		134 [29.8]	
**ANC visit (Times)(n=698)**				<0.001		<0.001		<0.001
	No ANC visit	108 [15.5]	15 [13.9]		64 [59.3]		11 [10.2]	
	1–3	211 [30.2]	43 [20.4]		167 [79.1]		35 [16.6]	
	4 or more	379 [54.3]	154 [40.6]		304 [80.2]		139 [36.7]	
**Deworming(n=698)**				0.001		0.026		<0.001
	No	441 [63.2]	154 [34.9]		350 [79.4]		139 [31.5]	
	Yes	257 [36.8]	58 [22.6]		185 [72.0]		46 [17.9]	
**Iron tablet or syrup(n= 698)**				0.001		0.003		<0.001
	No/Do not know	122 [17.5]	22 [18.0]		81 [66.4]		16 [13.1]	
	Yes	576 [82.5]	190 [33.0]		454 [78.8]		169 [29.3]	
**Frequency of reading newspaper or magazine(n=698)**				<0.001		0.080		<0.001
	Not at all	497 [71.2]	113 [22.7]		370 [74.4]		99 [19.9]	
	Less than once a week	154 [22.1]	75 [48.7]		128 [83.1]		67 [43.5]	
	At least once a week	47 [6.7]	24 [51.1]		37 [78.7]		19 [40.4]	
**Frequency of watching television(n=698)**				<0.001		<0.001		<0.001
	Not at all	265 [38.0]	52 [19.6]		188 [70.9]		44 [16.6]	
	Less than once a week	169 [24.2]	45 [26.6]		135 [79.9]		40 [23.7]	
	At least once a week	264 [37.8]	115 [43.6]		212 [80.3]		101 [38.3]	
**Frequency of listening radio(n=698)**				0.002		0.027		0.002
	Not at all	163 [23.4]	38 [23.3]		113 [69.3]		32 [19.6]	
	Less than once a week	258 [37.0]	69 [26.7]		199 [77.1]		60 [23.3]	
	At least once a week	277 [39.7]	105 [37.9]		223 [80.5]		93 [33.3]	
**Size of baby(n=698)**				0.016		0.431		0.024
	Average	435 [62.3]	142 [32.6]		338 [77.7]		124 [28.5]	
	Small	134 [19.2]	27 [20.21]		97 [72.4]		23 [17.2]	
	Large	129 [18.5]	43 [33.3]		100 [77.5]		38 [29.5]	
**Place of delivery(n=698)**				<0.001		0.057		<0.001
	Home	396 [56.7]	95 [24.0]		293 [74.0]		81 [20.5]	
	Health facility	302 [43.3]	117 [38.7]		242 [80.1]		104 [34.4]	
**Mode of delivery(n=698)**				0.048		0.257		0.025
	Vaginal delivery	660 [94.6]	195 [29.5]		503 [76.2]		169 [25.6]	
	Caesarean section	38 [5.4]	17 [44.7]		32 [84.2]		16 [42.1]	

# The number of missing values may vary for each variable. The percentages presented are valid percentages. *MMF* minimum meal frequency, *MAD* minimum acceptable diet.

The health related characteristics of the mother-child pairs included in the study are shown in Table 2. More than a third (36.1%) of the mothers was first time mothers. A large proportion of mothers did not read newspapers (71.2%), did not watch television (38.0%), and did not listen to radio (23.4%). Almost one in five (19.2%) mothers perceived that their babies were smaller than average. Four in ten (43.3%) births occurred at health facilities remainder being born in the home.

### Types of food given to child by age

Table 3 presents the seven food groups recommended by the WHO and recorded in the NDHS 2011 survey. Overall, for children aged 6–23 months, grains and tubers were provided to the greatest number of children (90.7%). Only one in three (30.6%) children were provided with vitamin A rich foods. For most of the food categories, it is apparent that the biggest jump in consumption occurred between the 12-17th months.

**Table 3:** Infant feeding practices among 6–23 months children of Nepal by age in 2011

**Food groups**	**Age of child**			
	**6–11 Months (n=244) n [%]**	**12–17 Months (n=238) n [%]**	**18–23 Months (n=216) n [%]**	**6–23 Months (N=698) n [%]**
1. Grains, roots and tubers	193 [79.1]	233 [97.9]	207 [95.8]	633 [90.7]
2. Legumes and nuts	99 [40.6]	132 [55.5]	114 [53.0]	345 [49.5]
3. Dairy products	117 [48.0]	121 [50.8]	114 [52.8]	352 [50.4]
4. Flesh food	18 [7.4]	52 [21.8]	56 [25.9]	126 [18.1]
5. Eggs	22 [9.0]	23 [9.7]	22 [10.2]	67 [9.6]
6. Vitamin A rich fruits and vegetables	41 [16.8]	93 [39.1]	82 [28.0]	216 [30.6]
7. Other fruits and vegetables	33 [13.5]	64 [26.9]	76 [35.3]	173 [24.8]

### Complementary feeding indicators

**Figure 1:**
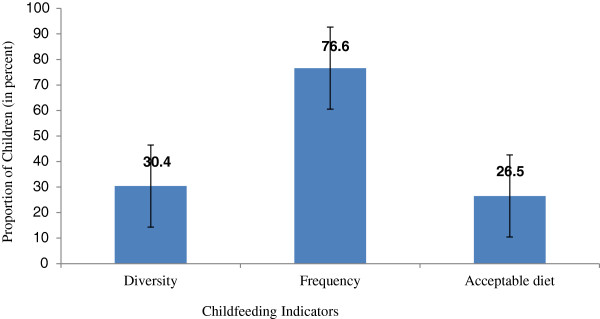
Prevalence of young child feeding practices among children of 6–23 months, Nepal 2011.

Figure 1 shows the proportion of children by the recommended complementary feeding indicators. Among children aged 6–23 months, only 30.4% of children met the requirements for minimum dietary diversity while, 76.6% met the dietary frequency and 26.5% met the minimum acceptable diet. Table 4 provides a more detailed account of the complementary feeding practices based on the age and the breastfeeding status of the children using the WHO guideline [7,15]. The proportion of children aged 6–11 months [17.6%, 95% CI (12.82%, 22.38%)] who received minimum dietary diversity was significantly lower than that of children aged 12–17 months [36.6%, 95% CI (30.48%, 42.72%)] and children aged 18–23 months [38.0%, 95% CI (31.53%, 44.47%)]. This trend is similar for minimum meal frequency and minimum acceptable diet, suggesting younger children (6–11 months) were the most vulnerable for not meeting these recommended infant feeding practices. For breastfed children (N=669), the proportion of children who were provided with minimum acceptable diet was 27.4%.

**Table 4:** Age wise disaggregation of infant feeding practices, Nepal 2011

**Age categories**	**Provided**	% (95% CI)*
**Minimum dietary diversity**		
Minimum dietary diversity for 6–11 months (n=244)	43	17.6 (12.82–22.38)
Minimum dietary diversity for 12–17 months (n=238)	87	36.6 (30.48–42.72)
Minimum dietary diversity for 18–23 months (n=216)	82	38.0 (31.53–44.47)
**Minimum dietary diversity for 6–23 months (N= 698)**	**212**	**30.4 (26.99-33.81)**
**Minimum meal frequency**		
Minimum meal frequency for 6–8 months breastfed (n=133)	88	66.2 (58.16–74.24)
Minimum meal frequency for 9–23 months breastfed (n=536)	421	78.5 (75.02–81.98)
Minimum meal frequency for 6–23 months non breastfed (n=29)	26	89.7 (78.64–100)
Minimum meal frequency for 6–23 months breastfed (n=669)	509	76.1 (72.87–79.33)
Minimum meal frequency for 6–11 months (all) (n=244)	169	69.3 (63.51–75.09)
Minimum meal frequency for12–17 months (all) (n= 238)	193	81.1 (76.13–86.07)
Minimum meal frequency for 18–23 months (all) (n=216)	173	80.1 (74.78–85.42)
**Minimum meal frequency for 6–23 months all (N=698)**	**535**	**76.6 (73.46–79.74)**
**Minimum acceptable diet**		
Minimum acceptable diet for breastfed children (n=669)	183	27.4 (24.02–30.78)
Minimum acceptable diet for non-breastfed children (n=29)	2	6.9 (N/A)
Minimum acceptable diet 6–11 months (all) (n=244)	41	16.8 (12.11–21.49)
Minimum acceptable diet 12–17 months (all) (n=238)	77	32.4 (26.45–38.35)
Minimum acceptable diet 18–23 months (all) (n= 216)	67	31.0 (24.83–37.17)
**Minimum acceptable diet for all children (6–23 months) (N=698)**	**185**	**26.5 (23.23–29.77)**

*N/A* not applicable for upper and lower limits of the proportion. *CI* confidence interval. *Proportions under the same section with a different superscript letter are significantly different (P<0.05).

### Factors associated with providing recommended complementary feeding practices

The factors associated with the minimum meal diversity are shown in Table 5. The age of the mother during pregnancy, development region, mother’s education, and father’s education were significantly associated with providing the minimum dietary diversity after controlling for other predictors in the model. It was found that children whose mothers were older, aged 35 or above at pregnancy, were more likely [aOR 2.546; 95% CI (1.042-6.223)] to be fed with diversity foods and hence, meet the minimum dietary diversity than those children whose mothers were 15–19 years at pregnancy. Children whose mothers were well educated and had a secondary level education [aOR 2.634; 95% CI (1.606-4.318)] or higher education [aOR 3.246; 95% CI (1.423-7.403)] were more likely to meet the minimum dietary diversity compared to children whose mothers did not have any formal education, indicating an increase in the odds of providing a diversity of foods with an increase in education level. Similarly, compared to no education, as the education level of the fathers increased, the children were more likely to get the recommended diversity of food -primary education [aOR 2.613; 95% CI (1.216-5.616)], secondary [aOR 4.278; 95% CI (2.035-8.992)] or higher [aOR 4.648; 95% CI (1.866-11.578)]. Children from the Mid-western region were less likely [aOR 0.451; 95% CI (0.258-0.787)] to be provided with the diversity food than children from the Eastern region; however, no differences were found between the Eastern and other regions.

**Table 5:** Determinants of meeting minimum dietary diversity among 6–23 months children, Nepal 2011 (N=698)

**Factor**	**Total** **N [%]**	**MDD** **n [%]**	**Crude OR**	**95% CI**	**Adjusted OR**	**95% CI**
**Mother’s age at pregnancy (in years)**			p=0.066		p=0.024*	
15–19	78 [11.2]	22 [28.2]	1.00		1.00	
20–29	479 [68.6]	159 [33.2]	1.265	0.746–2.146	1.286	0.719–2.299
30–34	83 [11.9]	16 [19.3]	0.608	0.291–1.268	0.637	0.282–1.436
>=35	58 [8.3]	15 [25.9]	0.888	0.412–1.912	2.546	1.042–6.223
**Mother’s education**			p<0.001*		p<0.001*	
No education	288 [41.3]	46 [16.0]	1.00		1.00	
Primary	131 [18.8]	31 [23.7]	1.631	0.978–2.720	1.149	0.653–2.021
Secondary	236 [33.8]	111 [47.0]	4.672	3.113–7.010	2.634	1.606–4.318
Higher	43 [6.2]	24 [55.8]	6.645	3.369–13.110	3.246	1.423–7.403
**Father’s education**			p<0.001*		p=0.001*	
No education	135 [19.3]	11 [8.1]	1.00		1.00	
Primary	155 [22.2]	33 [21.3]	3.049	1.474–6.306	2.613	1.216–5.616
Secondary	334 [47.9]	131 [39.2]	7.275	3.708–13.998	4.278	2.035–8.992
Higher	74 [10.6]	37 [50.0]	11.273	5.237–24.267	4.648	1.866–11.578
**Development region**			p=0.003*		p=0.036*	
Eastern	165 [23.6]	62 [37.6]	1.00		1.00	
Central	157 [22.5]	41 [26.1]	0.587	0.365–0.945	0.727	0.420–1.257
Western	100 [14.3]	36 [36.0]	0.934	0.558–1.565	0.746	0.418–1.333
Mid-western	167 [23.9]	34 [20.4]	0.425	0.260–0.694	0.451	0.258–0.787
Far-western	109 [15.6]	39 [35.8]	0.926	0.560–1.530	1.025	0.580–1.811

*: statistically significant. *MDD* minimum dietary diversity, *CI* confidence interval, *OR* odds ratio. -2loglikelihood ratio: 726.296 df: 16, Independent variables entered in initial model: mother’s age at pregnancy, residence, development region, ecological region, highest education of mother, education of father, occupation of mother, ethnicity, religion, wealth index, type of cooking fuel, birth order, birth interval by month, timing of last pregnancy, anaemia level of mother, deworming during pregnancy, iron consumption during last pregnancy, Size of the child at birth, place of delivery, mode of delivery, frequency of reading newspaper/magazine, frequency of watching television, frequency of listening radio. (Only significant predictors reported in the table).

Table 6 presents the factors associated with providing the recommended meal frequency. Antenatal visits, development region, and ecological region were significantly associated with the recommended minimum meal frequency after controlling for other predictors in the model. The mothers who attended ANC visits were more likely to meet minimum meal frequency requirements. The mothers with four or more ANC visits [aOR 2.3; 95% CI (1.332-3.849)] and one to three ANC visits [aOR 2.5; 95% CI (1.437-4.301)] were more likely to provide the recommended minimum number of feeds to their children compared to mothers who did not attend any ANC visit. Similarly, children from the Mid-western region [aOR 0.437; 95% CI (0.248-0.772)] and the Far-western region [aOR 0.449; 95% CI (0.235-0.858] were more at the risk of not getting the recommended meal frequency as compared to the children from the Eastern region. Children whose family lived in the Hill region were more likely [aOR 1.996; 95% CI (1.297-3.073)] to be provided with the recommended meal frequency.

**Table 6:** Determinants of meeting minimum meal frequency among 6–23 months children, Nepal 2011 (N=698)

**Factor**	**Total** **N [%]**	**MMF** **n [%]**	**Crude OR**	**95% CI**	**Adjusted OR**	**95% CI**
**ANC visit (times)**			p<0.001*		p=0.002*	
No ANC visit	108 [15.5]	64 [59.3]	1.00		1.00	
1–3	211 [30.2]	167 [79.1]	2.609	1.571–4.335	2.486	1.437–4.301
4 or more	379 [54.3]	304 [80.2]	1.760	1.760–4.412	2.264	1.332–3.849
**Development region**			p=0.011*		p=0.004*	
Eastern	165 [23.6]	142 [86.1]	1.00		1.00	
Central	157 [22.5]	116 [73.9]	2.134	1.153–3.949	0.591	0.325–1.074
Western	100 [14.3]	79 [79.0]	0.978	0.560–1.709	0.702	0.351–1.403
Mid-western	167 [23.9]	117 [70.1]	1.300	0.682–2.479	0.437	0.248–0.772
Far-western	109 [15.6]	81 [74.3]	0.809	0.470–1.392	0.449	0.235–0.858
**Ecological region**			p=0.006*		p=0.006*	
Terai	278 [39.8]	197 [70.9]	1.00		1.00	
Mountain	137 [19.6]	105 [76.6]	1.394	0.841–2.165	1.559	0.909–2.615
Hill	283 [40.5]	233 [82.3]	1.916	1.284–2.859	1.996	1.297–3.073

*: statistically significant. *MMF* minimum meal frequency, *CI* confidence interval, *OR* odds ratio. -2loglikelihood ratio: 688.015, df:12, Independent variables entered in the initial model: type of cooking fuel, sex of household head, father’s education, education of mother, development region, ecological region, place of delivery, frequency of reading newspaper magazine, frequency of watching television, frequency of listening radio, deworming, iron tablet consumption during pregnancy, antenatal visit, anaemia of mother, timing of last pregnancy, and type of pregnancy. Note: Fathers occupation was not entered into model as there was 13/13 [100%] in one category of children met meal frequency. (Only significant predictors reported in the table).

The factors associated with minimum acceptable diet can be found in Table 7. It was found that ANC visits, mother’s age, mother’s education, and father’s education were the significant determinants of providing a minimum acceptable diet, after controlling for other predictors in model. Mothers who had attended four or more ANC [aOR 2.6; 95% CI (1.266-5.343)] were more likely to provide an acceptable diet than mothers who had no antenatal visit. The mothers who were 30–34 years at pregnancy were less likely [aOR 0.220; 95% CI (0.079-0.616)] to provide the recommended acceptable diet than the mothers who were >=35 years. The mothers who had a high school education were more likely [aOR 2.159; 95% CI (1.269-3.595)] to provide the minimum acceptable diet. Children whose fathers had secondary [aOR 3.874; 95% CI (1.742-8.615)] or higher level [aOR 4.324; 95% CI (1.668-11.212)] were more likely to be provided with the recommended acceptable diet as compared to the children whose fathers did not have any education.

**Table 7:** Determinants of meeting minimum acceptable diet among 6–23 months children, Nepal 2011 (N=698)

**Factor**	**Total** **N [%]**	**MAD** **n [%]**	**Crude OR**	**95% CI**	**Adjusted OR**	**95% CI**
**ANC visit (times)**			p<0.001*		p=0.003*	
No ANC visit	108 [15.5]	11 [10.2]	1.00		1.00	
1–3	211 [30.2]	35 [16.6]	1.754	0.852–3.608	1.349	0.632–2.879
4 or more	379 [54.3]	139 [36.7]	5.107	2.646–9.858	2.601	1.266–5.343
**Mother’s age at pregnancy (in years)(n=698)**			p=0.018*		P=0.024*	
>=35	58 [8.3]	12 [20.7]	0.708	0.315–1.590	1.00	
15–19	78 [11.2]	21 [26.9]	1.00		0.485	0.189–1.244
20–29	479 [68.6]	141 [29.4]	1.123	0.661–1.938	0.567	0.256–1.257
30–34	83 [11.9]	11 [13.3]	0.415	0.185–0.930	0.220	0.079–0.616
**Mother’s education(n=698)**			p<0.001*		p=0.002*	
No education	288 [41.3]	40 [13.9]	1.00		1.00	
Primary	131 [18.8]	25 [19.1]	1.462	0.844–2.532	0.878	0.480–1.605
Secondary	236 [33.8]	100 [42.4]	4.559	2.989–6.953	2.159	1.269–3.595
Higher	43 [6.2]	20 [46.5]	5.391	2.715–10.706	1.780	0.775–4.090
**Father’s education**			p<0.001*		p=0.003*	
No education	135 [19.3]	9 [6.7]	1.00		1.00	
Primary	155 [22.2]	26 [16.8]	2.822	1.272–6.260	2.070	0.898–4.773
Secondary	334 [47.9]	118 [35.3]	7.648	3.751–15.595	3.874	1.742–8.615
Higher	74 [10.6]	32 [43.2]	10.667	4.708–24.166	4.324	1.668–11.212

*: statistically significant. *MAD* minimum acceptable diet, *CI* confidence interval, *OR* odds ratio. -2loglikelihood ratio: 682.338, df:13. Independent variables entered in the initial model: Mother’s age at pregnancy, place of residence, development region, ecological region, education of mother, education of father, occupation of mother, ethnicity, wealth index, type of cooking fuel, birth order, birth interval, timing of last pregnancy, anaemia of mother, antenatal visit, deworming, iron tablet consumption during pregnancy, frequency of reading newspaper/magazine, frequency of watching television and frequency of listening radio, size of the baby, place of delivery, and mode of delivery. (Only significant predictors reported in the table).

## Discussion

Like many developing countries, stunting and wasting have long been major nutritional problems in Nepal. The existing high proportion of under nutrition (41% stunting and 13% underweight among under five children) makes it complicated to reduce the under-five mortality in Nepal [4]. Nepal is a signatory of the millennium development goals (MDG) [16] and has achieved a significant reduction in child mortality [17]. It has committed to reduce child mortality by a further two-thirds by 2015 from the 1990 figures. Nepal has also committed to reduce extreme poverty and hunger [18]. Both of these goals are closely related to under nutrition and progress towards reaching these goals will be limited if the under nutrition is not reduced substantially. Not limited to these two goals, all other MDGs are directly or indirectly related to nutrition and are likely to worsen by under nutrition [19]. To reduce under nutrition adequate, safe and acceptable child feeding is essential. For this reason, WHO and UNICEF have recommended eight core infant feeding practices to be adopted [7]. To better promote such recommended practices, it is essential to demonstrate the evidence on the existing proportion of children reaching the dietary practice’s goals. This study reports the proportion and the determinants of receiving the recommended diets among the 6–23 months Nepalese children.

Three infant and young child feeding practices (minimum dietary diversity, minimum meal frequency and minimum acceptable diet) were assessed in this study based on the WHO recommendation and indicators [7,9,15]. It was found that only 30.4% of children received the recommended minimum dietary diversity, 26.5% received an acceptable diet and 76.6% received the recommended minimum meal frequency.The proportion of breastfed children provided with minimum acceptable diet was 27.4% which was slightly lower than the figure in 2006 (32%) [8]. In 2006 [8], 34% and 82% of the breastfed children received the minimum diversity and minimum meal frequency, respectively.

The prevalence of meeting these infant feeding practices varies across the countries in South Asia. The minimum diversity criteria reported in this study was higher than India (15.2%) but lower than Bangladesh (41.9%) and Sri Lanka (71.1%) [12]. Likewise, meal frequency was also less than in Bangladesh (81.1%) and Sri Lanka (88.3%). Current findings for attaining the minimum acceptable diet for children were also lower than previous studies in Nepal, Sri Lanka and Bangladesh [12]. It is difficult to determine how high the population percentage for infant and young child feeding practices would need to be in order to significantly eliminate current levels of under nutrition among children in Nepal. The WHO guidelines on infant feeding do not provide the baseline or the minimum standard that needs to be reached nor what percentage should be considered alarming for public health significance [7,15]. Logically, it is desirable that all young children (6–23 months) meet the recommended feeding practices.

To understand the low levels of dietary diversity and acceptable diet provided to Nepalese children, it is necessary to look at the food items provided to children. The majority of food items given to Nepalese children were from the grains, roots or tubers (Food Group 1) that are rich in carbohydrate (energy). Food items from the other six food groups were given to less than a half of the children. When diversity does not exist across the food items, it negatively affects the findings for minimum dietary diversity and acceptable diet.

A notable finding was a major change in diets across the various age groups that improved with age. This finding suggested that the youngest age group 6–11 months received the lowest proportion of food from all seven categories of food. This age group was least likely to meet the recommended meal frequency, meal diversity and acceptable diet standard than the older (12–23) children. It shows that 6–11 months children were even more at risk of under nutrition and micronutrient deficiency. The children of age group 6–23 months go through a reasonably rapid dietary transition from exclusive breastfeeding to complementary feeding. While changing the diet, they are vulnerable to diarrhoeal infections [20,21]. During this period, young children need more nutrition to overcome the adverse effect of such illness. Unfortunately, the current finding indicated that the children in this age group were not getting complementary foods as recommended by the WHO.

Nepal has achieved significant progress in reducing vitamin A deficiency disorders such as night blindness [16]. This achievement was mainly due to the high dose vitamin A supplementations that are provided twice a year to all under-five children. This supplementation is important given that currently only a third of the children in Nepal received vitamin A rich foods in their diet. A major public health strategy to increase vitamin A in the diet is to focus on dietary modification and increase consumption of vitamin A rich foods. Adequate vitamin A can be achieved through local foods but it requires careful attention to reach the need based on existing practices [22,23]. Miller et al. [24] quantified the dietary modification required if a child were to depend entirely on normal diet for vitamin A without any supplementation. They reported that a child in a developing country would need to increase the proportion of vegetable and fruit by about 10 folds to attain minimally adequate liver vitamin A storage. This 10-fold increase in vitamin A rich food may not be feasible to reach in Nepal in the short term. Therefore, the existing twice a year supplementation of high dose vitamin A (2,00,000 IU) should be maintained to prevent the reversal of progress made in controlling the vitamin A deficiency disorders so far [16,23,24].

Factors found to be associated with one of the three infant feeding practices were attending antenatal care visits, age of mothers, development region, and ecological region, education of mothers and education of fathers.

Antenatal visits during pregnancy are not very common in Nepal. Less than a half of all pregnant mothers meet the recommended four or more ANC visits [4,16]. Mothers who have attended at least four ANC visits may be more informed, have greater access to services and may be from a well off family, and thus more likely to be able to afford and provide a variety of foods to their children more frequently. This could explain why ANC was a significant determinant in meeting the recommended acceptable diet and meal frequency criteria.

The age of mothers at pregnancy was another determinant for dietary diversity and acceptable diet. Mothers who were pregnant at an older age (>=35 years) were more likely to provide diversity food and minimum acceptable diet than those mothers who were pregnant at the age of under 20 years. This could be due to the fact that older mothers may be more experienced/confident in feeding children and encouraging different types of foods than younger mothers [25].

Geographic differences are important in terms of determining the access to food and other services. The children from the Mid-western region were less likely to meet the minimum diversity and frequency criteria when compared to the children from Eastern region. Similarly, children from the Far -western region were less likely to get minimum frequency compared to the children from the Eastern region. While current results confirm the international findings for regional difference [11,13,14], it also reiterates an issue of insufficient child feeding in the Far-western and Mid-western part of Nepal. This could be a function of remoteness, geographic difficulties, less food production and higher levels of poverty in these regions [26]. Children as a general rule suffer greater disadvantage when living in impoverish conditions. Mid-western and Far-western regions have been suffering from the food insufficiency for a long time especially in the hilly and the mountainous areas [26,27]. Poverty is very high in the Mid-western and Far-western Hilly regions of Nepal. Deraniyagala [28] reported that the poverty levels in the Far-western and Mid-western hills were as high as three times than in the Eastern regions. Most of the Far-western and Mid-western regions have to depend on the limited amount of food provided by the government subsidies through the Nepal Food Corporation and international donors such as the World Food Program [26,27,29]. Unfortunately, such food aid (especially rice) has discouraged local food production such as potato, maize, barley and beans making these areas even more vulnerable for food insecurity than before. Historically there is a tension between the continuous push of imported rice and the option of encouraging locally cultivable food items such as potato, millet and barley [29]. This tension although not directly related to the health sector, it affects the food availability in the regions and cannot be ignored as a contributing factor to child under nutrition. Our finding further re-iterated the vulnerability of the children in the Mid-western and the Far-western development regions. This finding also suggests that policy makers and program managers of health and development assistance programs need to consider the regional differences when planning for further programs aimed at improving child nutrition in order to meet the MDG.

The education level of mothers and fathers has been consistently reported as the determinant of infant feeding [14,30]. This study also found similar results. A recent comparison of five Asian countries on infant feeding reported that mother’s education was a significant determinant of appropriate infant feeding [12]. Sri Lanka had the highest proportion of children meeting the infant feeding guidelines for diversity, frequency and acceptability; and this was linked to the higher education status of mothers and overall literacy [12]. The similar positive impact of education on feeding practices was also reported in a previous Nepalese study [8]. Educated mothers and fathers are more likely to understand the education message, more likely to be engaged in the paid work and may have a higher socioeconomic level which could positively impact on infant feeding practices.

There are a number of strengths of this study. This study is based on the NDHS which used internationally validated questionnaires and methodology [31]. The NDHS 2011 is nationally representative survey with a high response rate (>94%); therefore, the three infant feeding indicators reported in this study are generalisable for the entire country [8]. The current findings give an indication for future interventions and a benchmark for future comparisons. It should be noted that minimum acceptable diet was closely related to the minimum dietary diversity that was similar to the findings from other South Asian countries [12]. In future studies, dietary diversity could be considered as a simple proxy indicator for acceptable diet [12,32].

Like any other observational studies, this study has some limitations. The cross sectional nature of the study prevents it from developing causal inference. The information in DHS surveys is based on interviews and retrospective information. There is possibility that some of the responses might suffer from recall bias and socially desirable response. This study does not take account the multistage sampling during statistical analysis. This may cause less precise estimation of standard errors and confidence interval. Another limitation related to the three infant feeding practices included in this study is the quality and amount of food given. Although the definition of the indicators deals with the variety and frequency of food, it does not take account of the quality and amount of food provided. For instance, a child who has been provided all three recommended infant feedings criteria might still not have a nutritionally adequate diet. However, for the countries whose data rely on the DHS surveys, this is the best possible evidence on the infant feeding practices at this time [1,7].

### Implication for health programs to increase recommended infant feeding

Education has been an important facilitating factor for child nutrition and development worldwide. This finding of this study that mothers with education were providing the recommended infant feeds has important public health implication. While providing formal education is beyond the roles of health workers, it is feasible for health workers to educate mothers by counselling, and to provide skills to adopt the recommended infant feeding practices. Such educational interventions are also possible in Nepal through existing mothers group meetings, female community health volunteers, and outreach clinics including primary health care outreach clinics [33,34]. Vitamin A rich foods were provided to as few as one third of the children. This re-iterates the importance for the continuation of twice-a-year vitamin A supplementation in Nepal. Children from the Far and Mid-western development region were at most risk of not getting the recommended diets; and were at risk of suffering under nutrition. An appropriate mix of education and food supplements based on local resources could be a feasible option to increase recommended infant feeding practices and reduce under nutrition [5,35]. However, program managers should be careful that food supplementation does not create dependency, and most importantly, does not displace the local food production system [26]. The current findings showed that all children in the 6–23 month group were at the risk of not meeting the recommended feeding goals but it was the younger age group; the 6–11 months, who were the most vulnerable. Therefore, mothers with 6–11 month old children should be given special attention in designing education programs that promote the recommended child feeding practices while implementing additional nutritional support programs [26]. This study suggests that there is a need for future intervention studies directed at improving the infant feeding practices in Nepal to aid in reaching the MDG and to reduce extreme poverty and hunger. Intervention studies would provide a greater insight and suggest the most appropriate intervention that works in increasing the proportion of children meeting the WHO recommended feeding practices.

## Conclusions

The study revealed that a high proportion (>90%) of children received tuberous foods. Less than one third (30.6%) of the children were getting vitamin A rich fruits and vegetables. Children were getting energy rich foods but not all of the required nutrients. Only one in three children was meeting the recommended dietary diversity and acceptable diet. A comparatively higher proportion (76.6%) of children met the recommended minimum meal frequency. The age of mother during pregnancy, the development region, mother’s education, and father’s education were the significant determinants of achieving the recommended dietary diversity. ANC visits, and the place where the family lives (development region, and ecological region) were significant determinants impacting on meeting the recommended meal frequency. ANC visits and education of mothers were significant determinants of meeting the recommended minimum acceptable diet. An appropriate mix of educational intervention and supplementation could be feasible option to improve infant feeding practices in Nepal with a special focus on the Far and Mid-western region. There is a need for future intervention studies for improving the infant feeding practices.

## Competing interests

The authors declare that this study does not have any financial support. The author also declares no conflict of interest in relation to academic, religious or political aspects. This work is part of VK’s MPH dissertation.

## Authors’ contributions

VK conceived the study, performed statistical analysis, and wrote the manuscript. KS and YZ supervised the analysis and interpretation of results; and contributed in revision of the manuscript. All of the authors agreed on the final version of the manuscript.

## Authors’ information

VK holds an MPH degree. He has been working in child health programs in Nepal for more than five years. Newborn care and child nutrition is the focus of his work in Nepal and MPH studies. Yun Zhao is a senior lecturer in the School of Public Health and teaches in the postgraduate programs. She has an MSc and PhD in statistics. Kay Sauer is a senior lecturer in the School of Public Health and coordinates the MPH/DrPH programs. She has an MSc and PhD in Behavioural Sciences.

## Pre-publication history

The pre-publication history for this paper can be accessed here:

http://www.biomedcentral.com/1471-2431/13/131/prepub
